# Acetylation modification in the regulation of macroautophagy

**DOI:** 10.1007/s44307-024-00027-7

**Published:** 2024-06-07

**Authors:** Li Huang, Hongwei Guo

**Affiliations:** 1 Shenzhen Branch, Guangdong Laboratory of Lingnan Modern Agriculture, Key Laboratory of Synthetic Biology, Ministry of Agriculture and Rural Affairs, Agricultural Genomics Institute at Shenzhen, Chinese Academy of Agricultural Sciences, Shenzhen, 518120 China; 2 Shenzhen Key Laboratory of Plant Genetic Engineering and Molecular Design, Institute of Plant and Food Science, School of Life Sciences, Southern University of Science and Technology, Shenzhen, 518055 China

**Keywords:** Acetylation, Deacetylation, Lysine, Autophagy, Post-translational modification

## Abstract

Macroautophagy, commonly referred to as autophagy, is an evolutionarily conserved cellular process that plays a crucial role in maintaining cellular homeostasis. It orchestrates the delivery of dysfunctional or surplus cellular materials to the vacuole or lysosome for degradation and recycling, particularly during adverse conditions. Over the past few decades, research has unveiled intricate regulatory mechanisms governing autophagy through various post-translational modifications (PTMs). Among these PTMs, acetylation modification has emerged as a focal point in yeast and animal studies. It plays a pivotal role in autophagy by directly targeting core components within the central machinery of autophagy, including autophagy initiation, nucleation, phagophore expansion, and autophagosome maturation. Additionally, acetylation modulates autophagy at the transcriptional level by modifying histones and transcription factors. Despite its well-established significance in yeast and mammals, the role of acetylation in plant autophagy remains largely unexplored, and the precise regulatory mechanisms remain enigmatic. In this comprehensive review, we summarize the current understanding of the function and underlying mechanisms of acetylation in regulating autophagy across yeast, mammals, and plants. We particularly highlight recent advances in deciphering the impact of acetylation on plant autophagy. These insights not only provide valuable guidance but also inspire further scientific inquiries into the intricate role of acetylation in plant autophagy.

## Introduction

Macroautophagy (hereafter referred to as autophagy) is a conserved degradative mechanism that sequesters cytoplasmic cargos into double-membrane vesicles called autophagosomes. These vesicles subsequently fuse with vacuoles (in yeasts and plants) or lysosomes (in animals) to eventually break down their contents (Li and Vierstra 2012; Wen and Klionsky 2016; Marshall and Vierstra 2018; Qi et al. 2021). Under normal physiological conditions, autophagy operates at basal levels, contributing to cellular homeostasis. However, it exhibits dynamic responsiveness to various cellular or environmental stimuli, such as nutrient starvation, energetic and metabolic stresses, hypoxia, oxidative stress, pathogen infections, and endoplasmic reticulum (ER) stress (Lum et al. 2005; Liang et al. 2007; Xiong et al. 2007; Bellot et al. 2009; Geeraert et al. 2010; Liu et al. 2012; Chen et al. 2015a; Zhang et al. 2016, 2021; Huang et al. 2019, 2024).

To date, more than 40 conserved AuTophaGy proteins (ATGs) have been identified in the core autophagy machinery across yeast (*Saccharomyces cerevisiae*), mammals, and plants (Wen and Klionsky 2016; Marshall and Vierstra 2018; Qi et al. 2021; Jeon et al. 2022). These proteins, along with their regulatory factors, assemble into distinct functional complexes: (1) the ATG1/ULK1 (unc-51-like kinase1) protein kinase complex initiates autophagy (Mizushima 2010; Suttangkakul et al. 2011; Qi et al. 2020; 2022); (2) the class III phosphatidylinositol 3-kinase (PI3K) complex mediates phagophore nucleation (Xie et al. 2015; Wen and Klionsky 2016; Qi et al. 2021); (3) the ATG9 complex facilitates membrane delivery to expanding phagophores (Zhuang et al. 2017; Kotani et al. 2018; Huang et al. 2024), (4) the ATG8/LC3 (microtubule associated protein 1 light chain 3)–phosphatidylethanolamine (PE) and ATG5–ATG12 conjugation systems drive phagophore expansion and autophagosome maturation (Ohsumi 2001; Fahmy and Labonté 2017; Qi et al. 2021); and (5) the soluble N-ethylmaleimide-sensitive factor attachment protein receptor (SNARE) complex regulates autophagosome-lysosome fusion (Nair et al. 2011; Marshall and Vierstra 2018). The orchestrated interplay of these complexes contributes to the occurrence of autophagy (Li and Vierstra 2012; Huang et al. 2023, 2024).

Emerging evidence underscores the dynamical regulation of autophagy by protein acetylation across various stages, with a specific focus on ATG proteins and their regulators. Protein acetylation, a highly conserved process, exerts critical regulatory control over cellular processes. It occurs in two distinct forms, namely N^α^-terminal (Nt) acetylation and lysine acetylation (Choudhary et al. 2014; Drazic et al. 2016; Aksnes et al. 2019; Xia et al. 2022). Nt acetylation is an irreversible process that transfers the acetyl group of acetyl-CoA to the N^α^-terminus of a protein and mainly occurs co-translationally or post-translationally. In contrast, lysine acetylation is a reversible PTM that transfers the acetyl group to the ε-amino group of lysine residues in target proteins (Choudhary et al. 2009; 2014; Drazic et al. 2016). While acetylated lysine residues were initially and extensively studied in histones, reversible lysine acetylation also occurs on nonhistone proteins in the nucleus and cytoplasm. This process is catalyzed by lysine acetyltransferases (KATs) and deacetylases (KDACs) (Choudhary et al. 2009; 2014; Son et al. 2021). Thus far, KATs can be grouped into three major families: the GNAT (Gcn5-related N-acetyltransferase) family, the p300 (E1A binding protein 300)/CBP (CREB-binding protein) family, and the MYST (MOZ, Ybf2/Sas3, Sas2, and TIP60) family (Yuan et al. 2012; Bánréti et al. 2013; Choudhary et al. 2014; Son et al. 2021; Xia et al. 2022). Similarly, known KDACs can also divided into Rpd3/Hda1 family, Sirtuin/Sir2 family, and plant-specific HD-tuin/HDT families (Yang and Seto 2008; Bobde et al. 2022; Xia et al. 2022).

**Fig. 1:**
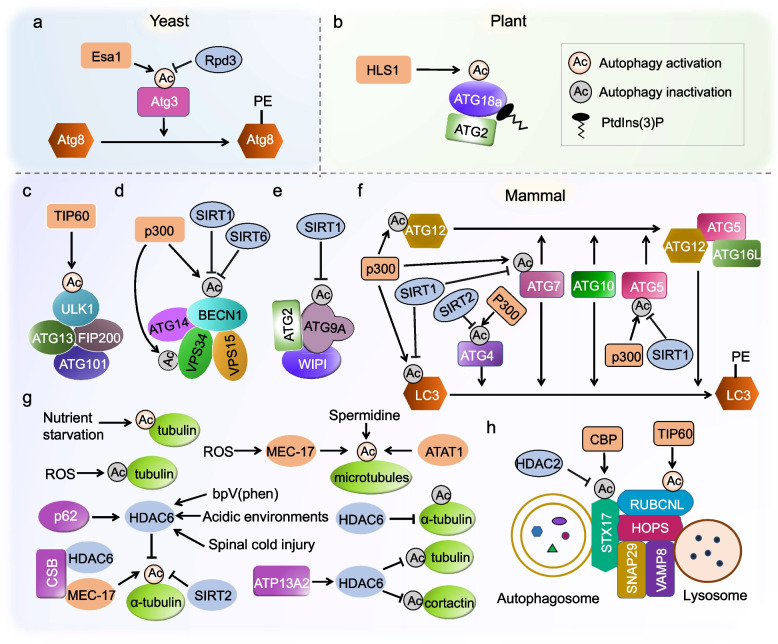
Regulation of autophagy by acetylation of the core autophagic machinery. **a** Esa1-mediated acetylation of Atg3 promotes ATG8 lipidation during nutrient scarcity. Conversely, Rpd3 catalyzes the deacetylation of Atg3. **b** In Arabidopsis, HLS1-mediated acetylation of ATG18a (an ortholog of yeast Atg18) positively regulates autophagy. **c** Acetylation of ULK1 by TIP60 promotes the activity of ULK1, thereby initiating autophagy. **d** In mammals, p300mediated acetylation of both BECN1 and VPS34 inhibit autophagy. Conversely, SIRT1 or SIRT6 mediated deacetylation of BECN1 achieves the opposite effect. **e** Acetylation of ATG9A prevents autophagy under ER stress conditions. Conversely, SIRT1-mediated ATG9A deacetylation leads to autophagy induction. **f** p300-mediated acetylation of ATG4B, ATG5, ATG7, and ATG12 inhibits autophagy. In contrast, SIRT1-mediated deacetylation of ATG5, ATG7 and LC3, as well as SIRT2-mediated deacetylation of ATG4B, enhance autophagy. **g** Acetylation of cytoskeleton proteins affects the activity of autophagy. **h** CBP and HDAC2 act as a molecular switch to modulate the acetylation of STX17. CBP-mediated acetylation of STX17 inhibits its activity, while HDAC2-mediated deacetylation of STX17 promotes the formation of the STX17-SNAP29-VAMP8 complex under starvation conditions. This complex is essential for autophagosome-lysosome fusion. Additionally, TIP60-mediated acetylation of RUBCNL facilitates HOPS complex recruitment, thereby promoting autophagosome maturation. Ac, acetylation; ATAT1, -tubulin acetyltransferase 1; ATG, autophagy-related; ATP13A2, ATPase cation transporting 13A2; BECN1, beclin 1; CSB, cockayne syndrome group B; bpV(phen), potassium bisperoxo (1,10-phenanthroline)oxovanadate; Esa1, essential SAS2-related acetyltransferase 1; FIP200, family-interacting protein of 200 kDa; HDAC6, histone deacetylase 6; LC3, microtubule-associated protein 1 light chain 3; MEC-17/TAT-1, -tubulin acetyltransferase-1; p300, E1A binding protein 300; p62/SQSTM1, sequestosome 1; PE, phosphatidylethanolamine; PtdIns(3)P, phosphatidylinositol 3-phosphate; ROS, reactive oxygen species; Rpd3, reduced potassium dependency-3; RUBCNL/Pacer, rubicon like autophagy enhancer; SIRT1, sirtuin 1; SIRT2, sirtuin 2; SIRT3, sirtuin 3; SIRT6, sirtuin 6; TIP60, HIV-1 Tat interactive protein 60 kD; ULK1, unc-51-like kinase1; VPS15/PIK3R4, phosphoinositide 3-kinase regulatory subunit 4; VPS34/PIK3C3, phosphatidylinositol 3-kinase catalytic subunit type 3

**Fig. 2:**
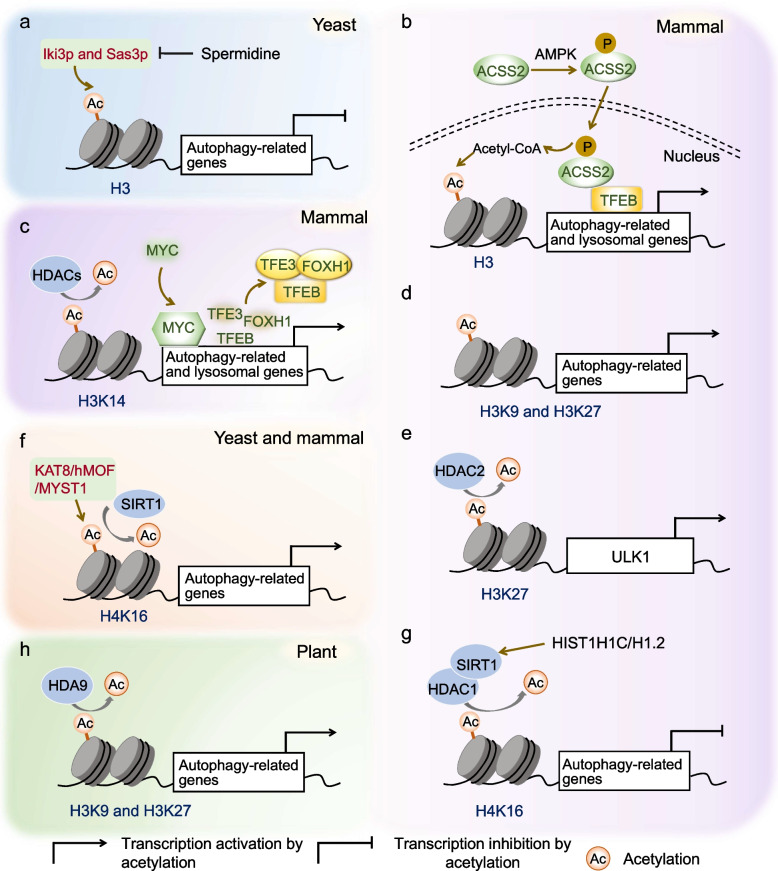
Epigenetic regulation of autophagy by histone acetylation. **a** In ageing yeast, spermidine inhibits the activity of HATs, such as Iki3p and Sas3p. This inhibition leads to decreased acetylation of H3 and upregulation of autophagyrelated genes. **b** Upon glucose deprivation, AMPK phosphorylates ACSS2, promoting its nuclear translocation. In the nucleus, ACSS2 binds to TEEB and locally produces acetyl-CoA for H3 acetylation in the promoter regions of autophagy-related and lysosomal genes, thereby activating gene expression. **c** The MYC protein cooperates with HDACs, specifically HDAC2, to suppress the transcription of autophagic and lysosomal. This suppression occurs through modulation of H3K14 acetylation and the occupancy of transcription factors TFEB, TFE3, and FOXH1 in the promoter regions of these genes. **d** Acetylation of H3K9 and H3K27 activates the expression of autophagy-related genes. **e** Treatment with short-chain fatty acids induces HDAC2 inhibition, leading to increased H3K27ac in the ULK1 promoter. Consequently, ULK1 transcription is upregulated. **f** Reduction of H4K16 acetylation, either through downregulation of KAT8/hMOF/MYST1 or deacetylation of H4K16 mediated by SIRT1, results in transcriptional repression of autophagy-related genes. **g** Overexpression of histone HIST1H1C/H1.2 upregulates SIRT1 and HDAC1, leading to reduced acetylation of H4K16. This reduction promotes the transcription of autophagy-related genes. **h** In Arabidopsis, HAD9-mediated histone deacetylation of H3K9 and H3K27 suppresses the expression of autophagic genes. ACSS2, acetyl-CoA synthetase 2; ATG, autophagy-related; Elp1/Iki3p, Elongator Acetyltransferase Complex Subunit 1; FOXH1, Forkhead Box H1; H3, histone 3; H4, histone 4; HATs, acetyltransferases; HDACs, deacetylases; HDA9, HISTONE DEACETYLASE9; K, lysine; KAT8, lysine acetyltransferase 8; MYC, BHLH transcription factor; Sas3p, the catalytic subunit of NuA3 HAT complex; SIRT1, sirtuin 1; TFEB, transcription factor EB; TFE3, Transcription Factor Binding to IGHM Enhancer 3; ULK1, unc-51-like kinase1

**Fig. 3:**
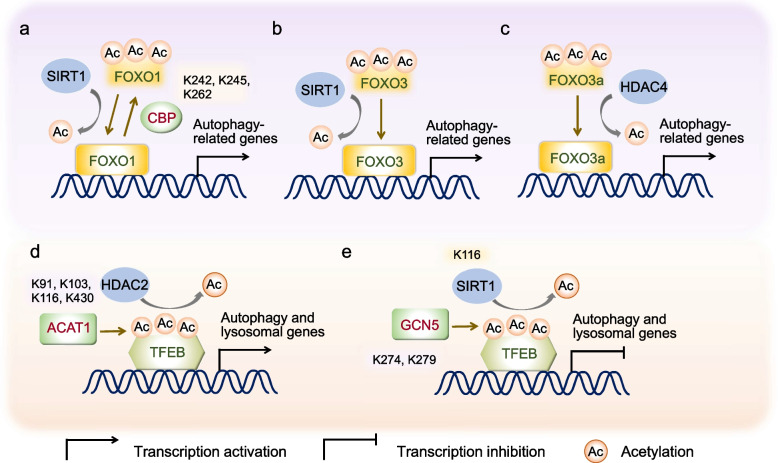
Regulation of autophagy by acetylation of transcription factors. **a** CBP-mediated acetylation of FOXO1 at K242, K245, and K262 attenuates its transcriptional activity, leading to down-regulation of autophagy-related genes. Correspondingly, the acetylation of FOXO1 can be reversed by SIRT1 in cardiac myocytes. **b** SIRT1 deacetylates FOXO3 to transcriptionally inhibiting autophagy in skeletal muscle. **c** Under Angiotensin II treatment, HDAC4-mediated FOXO3a deacetylation promotes autophagy at transcriptional level, which in turn promotes vascular inflammation. **d** In SAHA-treated cells, enhanced TFEB acetylation at K91, K103, K116, and K430 catalyzed by ACAT1 increases its transcriptional activity. Consequently, autophagy and lysosomal genes are upregulated. Notably, this acetylation can be reversed by HDAC2. **e** Acetylation of TFEB at K274 and K279 by GCN5 disrupts its DNA binding, resulting in the inhibition of transcription for autophagy and lysosomal genes. Conversely, SIRT1-mediated deacetylation of TFEB at K116 transcriptionally activating the expression of its downstream targets. ACAT1, acetyl-coenzyme A acetyltransferase 1; CBP, CREB binding protein; FOXO1, Forkhead Box O1; FOXO3, Forkhead Box O3; GCN5, general control non-repressed protein 5; HDAC2, histone deacetylase 2; HDAC4, histone deacetylase 4; K, lysine; SIRT1, sirtuin 1; TEEB, transcription factor EB

In this review, we discuss the pivotal regulatory roles of lysine acetylation by directly modulating core proteins involved in the central machinery of autophagy (Fig. 1; Table 1). We also summarize the recent advances in the impact of acetylation and its associated KATs and KDACs on the transcriptional regulation of autophagy-related genes (Figs. 2 and 3; Table 2). Furthermore, we highlight recent advancements in our understanding of lysine acetylation within the context of plant autophagy.

**Table 1:** List of acetylation of ATG proteins or autophagy-related proteins in autophagy regulation

Proteins	Modified sites	Acetyltransferases	Deacetylases	Activation or inhibition of autophagic activity	References
ULK1 (mammal)	K162, K606	TIP60	Unknown	Activation	Lin et al. 2012;
PIK3C3/VPS34 (mammal)	K29, K771, and K781	p300	Unknown	Inhibition	Su et al. 2017
Beclin 1 (mammal)	K430 and K437	p300	SIRT1 and SIRT6	Inhibition	Sun et al. 2015; Sun et al. 2018; Han et al. 2019
ATG9A (mammal)	Unknown	Unknown	SIRT1	Inhibition	Pehar et al. 2012; Pang et al. 2019
ATG18a (plant)	K323, K331, and K420	HLS1	Unknown	Activation	Huang et al. 2024
ATG4B (mammal)	K39	p300	SIRT2	Inhibition	Sun et al. 2022
Atg3 (yeast)	K19, K48, K183	Esa1	Rpd3	Activation	Yi et al. 2012
ATG5, ATG7, and LC3 (mammal)	Unknown	p300	SIRT1	Inhibition	Lee et al. 2008; Lee and Finkel 2009
ATG12 (mammal)	Unknown	p300	Unknown	Inhibition	Lee and Finkel 2009
LC3 (mammal)	K49 and K51	Unknown	Unknown	Inhibition	Huang et al. 2015; Liu and Klionsky 2015
Tubulin (mammal)	K40	Unknown	Unknown	Activation	Geeraert et al. 2010
Tubulin/cortactin (mammal)	Unknown	Unknown	HDAC6	Inhibition	Bonet-Ponce et al. 2016; Wang et al. 2019
Tubulin (mammal)	K40	Unknown	HDAC6	Inhibition	Li et al. 2018
-tubulin (mammal)	Unknown	MEC-17	HDAC6 and SIRT2	Activation	Chen et al. 2015b; Jiang et al. 2018; Majora et al. 2018; Esteves et al. 2019; Yang et al. 2019; Liang et al. 2020; Zheng et al. 2020
Microtubule (mammal)	Unknown	MEC-17/-TAT-1 and ATAT1	Unknown	Activation	Mackeh et al. 2014; Phadwal et al. 2018; Nowosad et al. 2021
STX17 (mammal)	K219 and K223	CBP	HDAC2	Inhibition	Shen et al. 2021
RUBCNL/Pacer (mammal)	K483, K523, K533, K573, and K633	TIP60	Unknown	Activation	Cheng et al. 2019

**Table 2:** List of protein acetylation in transcriptional regulation of autophagy

Types	Proteins	Modified sites	Acetyltransferases	Deacetylases	Transcriptional activation or inhibition	References
Histones	H3 (yeast)	K9, K14, and K18	Iki3 and Sas3	Unknown	Inhibition	Eisenberg et al. 2009
	H3 (mammal)	Unknown	Unknown	Unknown	Activation	Li et al. 2017
	H3 (mammal)	K14	Unknown	HDAC2	Activation	Annunziata et al. 2019
	H3 (mammal)	K9 and K27	Unknown	Unknown	Activation	Bhattacharjee et al. 2018; Qin et al. 2024
	H3 (mammal)	K27	Unknown	HDAC2	Activation	Du et al. 2021; Ma and Wang 2022
	H3 (plant)	K9 and K27	Unknown	HDA9	Activation	Chen et al. 2016; Yang et al. 2020a, b
	H4 (yeast and mammal)	K16	hMOF/KAT8/MYST1	SIRT1	Activation	Füllgrabe et al. 2013
	H4 (mammal)	K16	Unknown	SIRT1 and HDAC1	Inhibition	Wang et al. 2017
Transcription factors	FOXO1(mammal)	K242, K245, and K262	CBP	Unknown	Inhibition	Matsuzaki et al. 2005; Hariharan et al. 2010
	FOXO1(mammal)	Unknown	Unknown	SIRT1	Inhibition	Hariharan et al. 2010
	FOXO3 (mammal)	Unknown	Unknown	SIRT1	Inhibition	Mammucari et al. 2007
	FoxO3a (mammal)	Unknown	Unknown	HDAC4	Inhibition	Yang et al. 2018
	TFEB (mammal)	K91, K103, K116 and K430	ACAT1	HDAC2	Activation	Zhang et al. 2018
	TFEB (mammal)	K116	Unknown	SIRT1	Inhibition	Bao et al. 2016
	TFEB (mammal)	K274, and K279	GCN5	Unknown	Inhibition	Wang et al. 2020

## Acetylation modification of the core proteins in the central autophagy machinery

Protein acetylation plays a crucial role in directly modifying components of the core autophagy machinery that function at different stages. These stages include autophagy initiation, membrane delivery and nucleation, phagophore expansion, and autophagosome-lysosome fusion steps (McEwan and Dikic 2011; Bánréti et al. 2013; Sun et al. 2021; Xu and Wan 2023). In the following summary, we outline the current knowledge on the regulatory impact of autophagy-related protein acetylation (Fig. 1; Table 1).

### Acetylation during autophagy initiation

Autophagy induction relies on the activation of ATG1/ULK1 kinase complex during starvation (Chan et al. 2007; Cheong et al. 2007). In mammals, the ULK1 (homolog of yeast Atg1) kinase complex, which includes ULK1, family-interacting protein of 200 kDa (FIP200; the functional ortholog of yeast Atg17), autophagy-related protein 13 (ATG13), and ATG101 (Hosokawa et al. 2009; Jung et al. 2009; Mercer et al. 2009), is responsible for this activation. The activity of ULK1 complex is primarily regulated by two regulators, mechanistic target of rapamycin complex 1 (MTORC1) and AMP-activated protein kinase (AMPK). These regulators modulate the phosphorylation status of ULK1 (Kim et al. 2011; Xie et al. 2015). Acetyltransferase also plays a crucial role in regulating ULK1. Under deprivation of growth factors, the acetyltransferase HIV-1 Tat interactive protein 60 kD (TIP60) becomes activated through phosphorylation mediated by glycogen synthase kinase 3 (GSK3). This activation results in direct acetylation of ULK1 at K162 and K606, enhancing its kinase activity and thereby inducing autophagy (Lin et al. 2012). Similarly, the GSK3*β*-TIP60-ULK1 pathway is essential for modulating autophagy during endoplasmic reticulum (ER) stress (Nie et al. 2016). Collectively, the activity of ULK1 can be synergistically regulated by acetylation and phosphorylation modifications during autophagy induction. Notably, in plants, the activity of ATG1 is strictly modulated by phosphorylation, possibly mediated by SNF1 KINASE HOMOLOG 10 (KIN10), a plant ortholog of the mammalian AMPK (Li et al. 2014; Chen et al. 2017). Given the functional conservation of ATG1 across species, it is plausible that plant ATG1 may also undergo regulation via acetylation, similar to observations in mammals.

### Acetylation during nucleation and membrane delivery

The PI3K complex plays essential roles in the nucleation of the phagophore, a critical step following autophagy initiation (Funderburk et al. 2010; Marshall and Vierstra 2018; Qi et al. 2021). In mammals, this complex comprises core proteins, including beclin 1 (BECN1, the ortholog of yeast Vps30/Atg6), phosphoinositide 3-kinase regulatory subunit 4 (PIK3R4/VPS15), phosphatidylinositol 3-kinase catalytic subunit type 3 (PIK3C3/VPS34), and ATG14 (Xie et al. 2015). Phosphorylation of BECN1 by casein kinase 1 gamma 2 (CK1γ2) is necessary for its subsequent acetylation at K430 and K437, mediated by p300 in human cells (Sun et al. 2015). Consequently, acetylated BECN1 promotes its interaction with Run domain Beclin-1-interacting and cysteine-rich domain-containing protein (RUBCN/Rubicon), a negative regulator of VPS34 and autophagy (Matsunaga et al. 2009; Zhong et al. 2009). This interaction suppresses autophagy activation (Sun et al. 2015). In contrast, deacetylation of BECN1, mediated by SIRT1 or SIRT6, has the opposite effect (Sun et al. 2015; Sun et al. 2018; Han et al. 2019). Moreover, acetylation also regulates the activation of the lipid kinase VPS34. In human cells, p300 specifically acetylates VPS34 at K29, K771, and K781. The acetylation of VPS34 at different sites inhibits its lipid kinase activity through distinct molecular mechanisms (Su et al. 2017). For instance, acetylation of VPS34 at K29 disrupts its association with BECN1, while acetylation at K771 hinders VPS34-phosphatidylinositol (PI) interaction (Su et al. 2017). Thus, p300-mediated acetylation of both BECN1 and VPS34 inhibits autophagy in mammals. Additionally, mass spectrometry analysis in HeLa cells has identified acetylation of PIK3R4 at K951 (Weinert et al. 2013). Further exploration is warranted to uncover the contribution of PIK3R4 acetylation to autophagy regulation.

During the process of autophagy, the transmembrane protein ATG9 plays a pivotal role by forming a conserved complex with its partners, including ATG2 and ATG18/WIPI1/2. This complex facilitates the delivery of membranes, which is crucial for autophagosome formation (Xie et al. 2015; Marshall and Vierstra 2018; Qi et al. 2021). In eukaryotes, ATG9 is responsible for transporting lipids to the developing phagophore, a precursor structure for autophagosomes. The recycling of ATG9 is tightly regulated by several key players, including ATG1/ULK1 kinase, ATG2, and ATG18 (Reggiori et al. 2004; Young et al. 2006; Zhuang et al. 2017). In human cells, the acetylation status of ATG9A (the ortholog of yeast Atg9) dynamically responds to acetyl-CoA levels within the ER lumen. Acetylated ATG9A acts as a negative regulator of autophagy specifically under ER stress condition, as demonstrated by Pehar et al. (2012). Conversely, SIRT1-mediated ATG9A deacetylation functions as a sensor of ER stress, triggering autophagy (Pang et al. 2019). These findings underscore the critical role of ATG9 acetylation dynamics in modulating autophagy under specific circumstances, providing insights into the delicate balance required for cellular adaptation and survival.

ATG9–ATG18 complex is conserved across eukaryotes and participate in autophagosome formation (Zhuang et al. 2017; Marshall and Vierstra 2018). In plant, ATG9 is indispensable for the trafficking of ATG18a, an ortholog of yeast Atg18, on the autophagosomal membrane in a phosphatidylinositol 3-phosphate (PtdIns(3)P)-dependent manner. Recently, Arabidopsis HOOKLESS1 (HLS1) has been identified as an acetyltransferase that acetylates ATG18a both in vivo and in vitro. Reduced acetylation of ATG18a via genetic mutations hinders the interaction between ATG2 and ATG18a, as well as the binding activity of ATG18a to PtdIns(3)P. Consequently, autophagy is suppressed in response to nutrient starvation (Huang et al. 2024). It will be an intriguing question to address whether and how ATG18a acetylation affects the trafficking of ATG9 vesicles. Beyond acetylation, ATG18a undergoes additional modifications, including phosphorylation and persulfidation upon infection by necrotrophic pathogens (Zhang et al. 2021) and during ER stress (Aroca et al. 2021), respectively. These diverse PTMs of ATG18a in distinct cellular contexts suggest a finely tuned regulatory network. Given that ATG18a is part of the ATG18 family (ATG18a–ATG18h) in Arabidopsis, further investigations are necessary to elucidate whether acetylation also governs other members of this family.

### Acetylation during phagophore expansion

Phagophore expansion involves two ubiquitin-like ligation systems. These systems comprise the ATG12–ATG5–ATG16 complex and ATG8/LC3–phosphatidylethanolamine (PE) (Ohsumi 2001; Li and Vierstra 2012). To assemble the ATG12–ATG5–ATG16 complex, ATG12 is conjugated to ATG5 through the action of the E1-like enzyme ATG7 and the E2-like enzyme ATG10. Subsequently, the ATG12-ATG5 conjugate interacts with ATG16, forming an autophagy elongation complex that provides the site for ATG8 lipidation (Fujita et al. 2008; Fahmy and Labonté 2017). Moreover, the conjugation of ATG8/LC3–PE requires the involvement of the E2-like enzyme ATG3, the ATG4 protease (ATG4B in mammals), and ATG7 (Xie et al. 2015; Fahmy and Labonté 2017).

Accumulating evidence suggests that the core components required for the formation of ATG8/LC3–PE ligation system are tightly regulated by acetylation modification (Lee et al. 2008; Lee and Finkel 2009; Yi et al. 2012; Yi and Yu 2012; Huang et al. 2015). In yeast, Atg3 serves as the target of histone acetyltransferase essential SAS2-related acetyltransferase 1 (Esa1). Acetylation of Atg3 at distinct lysine sites regulates specific autophagic processes, similar to the role of VPS34 in mammals (Yi et al. 2012; Su et al. 2017). Under conditions of nitrogen starvation, Esa1-mediated induction of Atg3 acetylation at K19 and K48 promotes the Atg3-Atg8 interaction, thereby enhancing autophagy. Atg3 acetylation at K183 mediated by Esa1 is crucial for its lipid-conjugating activity (Yi et al. 2012). Conversely, Atg3 deacetylation, resulting from the histone deacetylase reduced potassium dependency-3 (Rpd3), leads to autophagy inhibition (Yi et al. 2012). Notably, Esa1-mediated acetylation of Atg3 is conserved in mammals. The ortholog of yeast Esa1, KAT5/TIP60, can also regulate autophagy by acetylating ATG3, although the corresponding deacetylase remains unidentified (Yi and Yu 2012). In human cells, LC3 can be target by acetyltransferase p300 or its closely related CBP for acetylation at K49 and K51. This acetylation event suppresses autophagy by preventing the cytoplasmic redistribution of nuclear LC3 during starvation (Lee and Finkel 2009; Huang et al. 2015). In contrast, SIRT1-mediated deacetylation of LC3 has the opposite effect (Lee et al. 2008; Huang et al. 2015; Li et al. 2016). Deacetylation of LC3 at K49 and K51, catalyzed by SIRT1 in the nucleus, results in its translocation to the cytoplasm through binding to tumor protein 53-induced nuclear protein 2 (TP53INP2/DOR). This translocation, in turn, promotes the LC3-ATG7 interaction, ultimately activating autophagy in starved cells (Huang et al. 2015; Liu and Klionsky 2015). Additionally, acetylation modification can stabilize LC3 by inhibiting its proteasome-dependent degradation (Song et al. 2019). Furthermore, recent research has shed light on the regulation of ATG4B, an orthologue of yeast Atg4, through acetylation and deacetylation processes mediated by p300 and SIRT2, respectively (Sun et al. 2022). Acetylation of ATG4B at K49, catalyzed by p300, suppresses ATG4B activity and induction of autophagy. Conversely, SIRT2 activation induced by starvation upregulates the deacetylation of ATG4B at K49. This deacetylation enhances the interaction between ATG4B and pro-LC3, as well as LC3 lipidation, thus inducing autophagy (Sun et al. 2022).

In addition to LC3 and ATG4B, ATG5, ATG7, and ATG12 proteins are also modified by acetyltransferase p300 in mammalian cells (Lee and Finkel 2009). Knockdown of p300 enhances autophagy by reducing the acetylation of ATG5, ATG7, and ATG12, while overexpression of p300 suppresses starvation-induced autophagy (Lee and Finkel 2009). Moreover, p300 influences the acetylation of ATG7 through physical interaction with it in a nutrient-dependent manner (Lee and Finkel 2009). Conversely, Lee et al. (2008) identified that SIRT1 deacetylates ATG5 and ATG7, thereby stimulating autophagy by controlling the activity of these proteins. Together, p300 and SIRT1 act as molecular switches, tightly regulating the acetylation and deacetylation status of core ATG proteins essential for autophagosome formation. This dynamic balance ensures proper autophagy under both normal and starved conditions.

Furthermore, it is noteworthy that several core ATG proteins have additional roles beyond autophagy (Lee et al. 2012; Maskey et al. 2013; Schaaf et al. 2016). For instance, LC3 family proteins can regulate autophagy-unrelated processes such as membrane trafficking and growth (Schaaf et al. 2016). Moreover, ATG5 is necessary for DNA damage induced by anticancer drugs and promotes mitotic catastrophe independent of autophagy (Maskey et al. 2013). Another study has highlighted the involvement of ATG7 in cell cycle regulation by modulating the activity of the tumor suppressor p53 (TP53/p53), independent of its E1-like enzymatic activity required for LC3 lipidation during phagophore expansion (Lee et al. 2012). In Arabidopsis, the interactome of wild-type ATG5 and its autophagy-inactive mutant reveals acetylation within the plant ATG5 complex, suggesting functions beyond autophagy (Elander et al. 2023). Hence, investigating whether acetylation of these ATG proteins governs processes other than autophagy would be a worthwhile endeavor.

### Acetylation during autophagosome delivery and fusion

Following the formation of autophagosomes, these vesicles are transported along microtubules to the microtubule-organizing centre (MTOC), where lysosomes are typically concentrated in mammals (Monastyrska et al. 2009; Geeraert et al. 2010; Bánréti et al. 2013). Subsequently, autophagosomes fuse with lysosomes or vacuoles, leading to the degradation of autophagic substrates in eukaryotic cells (Xu and Wan 2023). The fusion process appears to be intricately regulated by several factors, including the soluble N-ethylamide-sensitive factor attachment protein receptor (SNARE) complex, RAB GTPases, and the homotypic fusion and vacuole protein sorting (HOPS) complex, as substantiated by accumulating evidence (Itakura et al. 2012; Jiang et al. 2014; Wang et al. 2016; Xu and Wan 2023). Furthermore, cytoskeleton proteins, such as α-tubulin and cortactin, assume pivotal roles in autophagy-lysosome fusion and the trafficking of autophagic vesicles (Köchl et al. 2006; Lee et al. 2010; Wang et al. 2019).

The reversible acetylation of α-tubulin directly modulates the stability and function of microtubules, thereby influencing autophagosome formation and the fusion of autophagosomes with lysosomes (Piperno et al. 1987; Monastyrska et al. 2009; Xie et al. 2010; Bánréti et al. 2013; Liu et al. 2019; Shu et al. 2023). Under nutrient-starved conditions, tubulin acetylation at K40 increases in both labile and stable microtubules, subsequently promoting c-Jun N-terminal kinase (JNK) phosphorylation and activation. Activated JNK then triggers the release of BECN1 from Bcl-2-BECN1 complexes and its recruitment to microtubules, ultimately stimulating autophagy (Geeraert et al. 2010). Additionally, reactive oxygen species (ROS) activate α-tubulin acetyltransferase-1 (αTAT-1/MEC-17) in human HeLa cells, leading to microtubule hyperacetylation. This acetylation, in turn, promotes autophagy and cell survival under stress (Mackeh et al. 2014). Intriguingly, p300 negatively regulates MEC-17 expression and is recruited to microtubules under stress, indirectly influencing tubulin acetylation (Mackeh et al. 2014). However, ROS-induced hyperacetylation of tubulin inhibits autophagosome-lysosomes fusion in rotenone-treated ARPE-19 cells, suggesting cell-type-specific regulation of autophagy by tubulin acetylation (Bonet-Ponce et al. 2016). In an in vitro prion system, spermine treatment increases microtubule acetylation, facilitating the selective autophagic degradation of prion aggregates by binding to the microtubule protein Tubulin beta-6 chain (Tubb6) (Phadwal et al. 2018). Recent research also highlights that α-tubulin acetyltransferase 1 (ATAT1)-mediated microtubule acetylation promotes autophagosome trafficking along microtubule tracks in glucose-deprived cells (Nowosad et al. 2021). The conservation of α-tubulin acetylation in autophagy regulation across plant species remains an open question.

Histone deacetylase 6 (HDAC6) functions as an α-tubulin deacetylase, finely controlling autophagosome maturation and autophagosome-lysosome fusion through selective autophagy pathways (Hubbert et al. 2002; Iwata et al. 2005; Lee et al. 2010). In mammalian cells, potassium bisperoxo (1,10-phenanthroline) oxovanadate [bpV(phen)] enhances the activity of HDAC6 and disrupts the fusion of acetylated microtubule-dependent autophagosomes with lysosomes, as well as the degradation of autophagosomes. This disruption occurs by interfering with the interaction between HDAC6 and sequestosome 1 (SQSTM1/p62) (Chen et al. 2015b). A study has demonstrated that elevated p62 levels maintain the deacetylase activity of HDAC6, resulting in reduced α-tubulin acetylation and stabilized microtubules. Consequently, this leads to autophagy inhibition and epithelial-mesenchymal transition in prostate cancer cells (Jiang et al. 2018). Moreover, the acetylation of α-tubulin may be regulated by the Cockayne syndrome group B (CSB) protein through its interaction with deacetylase HDAC6 and acetyltransferase MEC-17. Inhibition of HDAC6 enhances α-tubulin acetylation, improving autophagic function and rescuing the loss of subcutaneous fat in CSB-deficient mice. Additionally, it improves podocytes motility in diabetic nephropathy (Majora et al. 2018; Liang et al. 2020). Yang et al. (2019) found that acidic environments increase HDAC6 activity, which reduces the α-tubulin acetylation, leading to impaired autophagosome formation and cardiomyocyte injury. Furthermore, spinal cord injury upregulates HDAC6, which in turn increases α-tubulin deacetylation. This affects the stability of the microtubule system and results in the inhibition of autophagic flux (Zheng et al. 2020). Another tubulin deacetylase, human SIRT2, has also been identified and is interdependent with HDAC6 in tubulin deacetylation (North et al. 2003). In human cells, SIRT2 mediates the deacetylation of α-tubulin and Tau, suppressing autophagic vesicular traffic and cargo clearance in Parkinson’s and Alzheimer’s diseases, as mediated by HDAC6 (Esteves et al. 2019).

In certain cases, HDAC6-mediated deacetylation of α-tubulin plays a positive role in autophagosome maturation. For example, in human cells, the deficiency of heterogeneous nuclear ribonucleoprotein K (hnRNPK) leads to reduced α-tubulin acetylation at K40 due to increased HDAC6 activity, thus enhancing autophagosome-lysosome fusion (Li et al. 2018). Additionally, the polyamine transporter P5B-type ATPase ATP13A2 (ATPase cation transporting 13A2) significantly contributes to maintaining lysosomal homeostasis through an HDAC6-mediated mechanism. ATP13A2 recruits HDAC6 to lysosomes, where it deacetylates cortactin and tubulin, thereby facilitating autophagosome-lysosome fusion and promoting autophagy (Wang et al. 2019). These findings highlight the critical roles of dynamic acetylation modifications of microtubules and tubulin in autophagy regulation. Further research should explore the impact of tubulin acetylation at different stages of autophagy.

Acetylation has also been implicated in regulating SNARE and HOPS complexes, which are essential for autophagosome-lysosome fusion (Cheng et al. 2019; Shen et al. 2021a). STX17, a core autophagosomal SNARE protein, resides on the outer membrane of the completed autophagosomes and is indispensable for fusion with the lysosomes in mammals (Itakura et al. 2012). Recent research has revealed that STX17 acetylation, catalyzed by the acetyltransferase CREBBP/CBP and the deacetylase HDAC2, modulates its SNARE activity and autophagosome maturation (Shen et al. 2021a). When starved, CBP activity is inhibited, leading to deacetylation of STX17 at K219 and K223 within the SNARE domain (Shen et al. 2021a). Deacetylated STX17 interacts with synaptosome-associated protein 29 (SNAP29), forming the STX17-SNAP29-VAMP8 (vesicle-associated membrane protein 8) SNARE complex. In addition, STX17 deacetylation at K219 and K223 promotes recruitment of the HOPS complex to the autophagosomal membranes, further facilitating autophagosome-lysosome fusion (Shen et al. 2021a). In contrast, under nutrient deprivation, the acetyltransferase TIP60 mediates the acetylation of rubicon like autophagy enhancer (RUBCNL/Pacer). This acetylation promotes HOPS complex recruitment and enhances autophagosome maturation (Cheng et al. 2019; Cheng and Sun 2019). Although CBP and TIP60 exhibit opposite roles in both early and late stages of autophagy in mammals, their regulatory functions in yeast and plants remain an intriguing area for systematic exploration.

## Acetylation modification and transcriptional regulation of autophagy-related genes

Transcriptional regulation is a pivotal mechanism that enables cells to maintain autophagy activity (Füllgrabe et al. 2014; Seok et al. 2014; Yang et al. 2020a). In addition to directly regulating ATGs or autophagy-related proteins through acetylation, acetylation also plays crucial roles in modulating autophagy gene expression across yeast, mammals, and plants. Acetylation modifications of histones and transcription factors (TFs) orchestrate the delicate balance between autophagy activation and repression at the transcriptional level, ensuring cellular homeostasis. (Shu et al. 2023; Figs. 2 and 3; Table 2).

### Histone acetylation and autophagy

Histone acetylation is a precisely controlled process governed by histone acetyltransferases (HATs) and histone deacetylases (HDACs). This dynamic interplay is intrinsically linked with the epigenetic regulation of gene expression by influencing chromatin structure and function (Grunstein 1997; Füllgrabe et al. 2010). Within this regulatory framework, the acetylation of histone 3 (H3) and H4 plays a pivotal role in controlling autophagy at the transcriptional level (Jeon et al. 2022; Shu et al. 2023; Fig. 2; Table 2). However, whether this regulation leads to transcriptional repression or activation depends on the specific type and modified site of histones.

Histone H3 acetylation is implicated in the fine regulation of autophagy-related genes. In ageing yeast, the compound spermidine inhibits the activity of HATs, including Elongator Acetyltransferase Complex Subunit 1 (Elp1/Iki3p) and the catalytic subunit of NuA3 HAT complex (Sas3p). This inhibition leads to global histone H3 hypoacetylation, resulting in an upregulation of autophagy-related transcripts (Eisenberg et al. 2009). In mammalian cells, histone acetylation-mediated autophagy regulation plays a crucial role in tumor progression. Under conditions of glucose deprivation, the protein kinase AMPK facilitates the nuclear translocation of acetyl-CoA synthetase 2 (ACSS2) by phosphorylating it at serine659 (S659) (Li et al. 2017). ACSS2 then associates with the transcription factor EB (TEEB), locally producing acetyl-CoA for histone H3 acetylation within the promoter regions of autophagy and lysosomal genes. This process contributes to lysosomal biogenesis, autophagy, and ultimately, tumorigenesis (Li et al. 2017). In addition, the transcription factor MYC collaborates with HDACs, specifically histone deacetylase 2 (HDAC2), to epigenetically inhibit autophagy and lysosomal function. This occurs through modulating acetylation of histone H3 at lysine 14 (H3K14) in the promoter regions of autophagic and lysosomal genes. Furthermore, the occupancy of transcription factors TFEB, Transcription Factor Binding to IGHM Enhancer 3 (TFE3), and Forkhead Box H1 (FOXH1) in these promoter regions contributes to the regulatory mechanism (Annunziata et al. 2019).

In contrast, H3K27 acetylation (H3K27ac) consistently correlates with the upregulation of autophagy-related genes (Bhattacharjee et al. 2018; Du et al. 2021; Ma and Wang 2022). In human cells, the Epstein-Barr virus (EBV) oncoprotein EBNA3C activates the transcription of autophagy-related genes, particularly *ATG3*, *ATG5* and *ATG7*, by recruiting histone activation epigenetic marks, including H3K9ac and H3K27ac (Bhattacharjee et al. 2018). More recently, Qin et al. (2024) further demonstrated the positive effect of H3K9ac and H3K27ac on autophagy-related genes, reinforcing the importance of these acetylation marks in autophagy regulation. Correspondingly, the reduction of H3K27ac due to pyocyanin (PYO) treatment decreases the recruitment of H3K27ac to the promoter region of ULK1 and represses *ULK1* transcription (Du et al. 2021). Additionally, a recent study indicates that increased H3K27 levels are associated with gene transcription activation. In diabetic mice, treatment with short-chain fatty acids (SCFAs) leads to HDAC2 inhibition, which in turn promotes H3K27ac in the ULK1 promoter. This upregulates ULK1 transcription and enhances autophagy, ultimately attenuating renal fibrosis (Ma and Wang 2022).

Interestingly, autophagy induction is generally coupled with reduced H4K16ac. In mammalian cells, the histone acetyltransferase KAT8/hMOF/MYST1 and deacetylase sirtuin 1 (SIRT1) function as a molecular switch to modulate the acetylation status of H4K16. During autophagy induction, autophagy-related genes are transcriptionally repressed due to the reduction of H4K16ac through downregulation of KAT8/hMOF/MYST1 or direct deacetylation of H4K16. This establishes a negative regulatory feedback loop (Füllgrabe et al. 2013). However, in the context of diabetic retinopathy development, the overexpression of histone HIST1H1C/H1.2, an important variant of the linker histone H1, upregulates SIRT1 and HDAC1 to reduce the acetylation status of H4K16. Consequently, the decreased H4K16ac leads to increased transcription of *Becn1*, *Atg3*, *Atg5*, *Atg7*, and *Atg12* genes and upregulation of ATG proteins, thereby promoting autophagy in cultured retinal cell line (Wang et al. 2017).

Histone acetylation also plays a pivotal role in transcriptional regulation of autophagy in plant development and abiotic stress responses (Chen et al. 2016; Yang et al. 2020a, b). In Arabidopsis, the histone deacetylase HISTONE DEACETYLASE9 (HDA9), a member of the reduced potassium dependency 3 (RPD3) class, exerts negative control over the expression of autophagy-related genes, including *ATG2*, *ATG8e*, *ATG9*, and *ATG13* genes, during senescence. This control is achieved by catalyzing the deacetylation of H3K9 and H3K27 (Chen et al. 2016). Moreover, a recent study by Yang et al. (2020b) supports the significance of HDA9-mediated deacetylation in plant autophagy. The Arabidopsis transcription factor ELONGATED HYPOCOTYL5 (HY5) negatively regulates autophagy by recruiting HDA9 to catalyze histone deacetylation of H3K9 and H3K27 during light-to-dark conversion and nitrogen deprivation. This suppression results in the inhibition of *ATG5* and *ATG8e* expression (Yang et al. 2020b). However, the impact of alterations in the acetylation of other histone types on plant autophagy remains an area of ongoing investigation.

### Acetylation of transcription factors and autophagy

Beyond histone acetylation, several TFs have emerged as key regulators of autophagy through acetylation-mediated mechanisms (Matsuzaki et al. 2005; Mammucari et al. 2007; Zhang et al. 2018; Wang et al. 2020; Shu et al. 2023). Among these, Forkhead Box O (FOXO) TFs family members and TFEB have been extensively demonstrated to be closely associated with the transcription regulation of autophagy via acetylation (Bánréti et al. 2013; Sun et al. 2021; Jeon et al. 2022; Shu et al. 2023; Fig. 3; Table 2).

The FOXO family, which is evolutionarily conserved across species, includes FOXO1, FOXO3, FOXO4, and FOXO6 in mammals (Salih and Brunet 2008). Acetylation dynamically modulates the transcriptional activity of FOXOs, thereby fine-tuning the expression of genes involved in autophagy activation (Sengupta et al. 2009; Bertaggia et al. 2011; Brown and Webb 2018). For instance, CBP-mediated acetylation of FOXO1 at specific lysine residues (K242, K245, and K262) attenuates its transcriptional activity, leading to down-regulation of the target gene expression, including those implicated in autophagosome formation (Matsuzaki et al. 2005; Sengupta et al. 2009). In cardiac myocytes, SIRT1-induced deacetylation of FOXO1 promotes autophagy by upregulating the expression of autophagy-related genes under conditions of glucose deprivation (Hariharan et al. 2010). Additionally, acetylation of FOXO1 can also impact autophagy through a transcription-independent process (Zhao et al. 2010). When human cells are subjected to serum starvation or oxidative stress, cytosolic FOXO1 becomes acetylated due to dissociation from SIRT2. The resulting acetylated FOXO1 then interacts with ATG7, facilitating autophagy induction (Zhao et al. 2010). In addition to FOXO1, FOXO3 has also been shown to undergo deacetylation by SIRT1, a critical event for transcriptionally activating autophagy in skeletal muscle (Mammucari et al. 2007; Kume et al. 2010). Moreover, HDAC4-mediated deacetylation of FOXO3a regulates autophagy activation at the transcriptional level, subsequently promoting vascular inflammation in response to Angiotensin II (Ang II) treatment (Yang et al. 2018). These findings collectively highlight the critical significance of acetylation modifications on FOXO proteins in the transcriptional regulation of autophagy.

Similar to FOXOs, TFEB serves as another key regulator of autophagy and lysosome-related gene expression. Its activity is also governed by acetylation (Settembre et al. 2011). However, the impact of acetylation on the transcriptional activity of TFEB remains a subject of debate, as it is context-dependent. In cells treated with suberoylanilide hydroxamic acid (SAHA), TFEB acetylation at K91, K103, K116, and K430 is enhanced by acetyl-coenzyme A acetyltransferase 1 (ACAT1), resulting in increased transcriptional activity and subsequent promotion of the expression of autophagy- and lysosome-related genes. Conversely, HDAC2 can reverse this acetylation (Zhang et al. 2018). However, a recent study revealed that acetylation of TFEB at K274 and K279, mediated by histone acetyltransferase general control non-repressed protein 5 (GCN5), hinders its DNA binding ability, leading to the inhibition of autophagy and lysosome biogenesis (Wang et al. 2020). Furthermore, SIRT1-mediated deacetylation of TFEB at K116 enhances microglial degradation of fibrillar β-amyloid (fAβ) by transcriptionally activating the expression of downstream targets, ultimately reducing amyloid plaques deposition (Bao et al. 2016). Collectively, the role of TFEB acetylation in autophagy regulation is intricately linked to its transcriptional activity, which is controlled by distinct acetyltransferases or deacetylase under varying conditions.

In plant, an increasing number of TFs have been demonstrated to orchestrate the transcriptional regulation of *ATG* genes, particularly in response to adverse environmental conditions (Yang et al. 2020a; Li et al. 2023). However, the specific impact and mechanistic role of TF acetylation in governing plant autophagy remain an area of active investigation. Consequently, it is imperative to identify the specific KATs and KDACs associated with these TFs and explore the underlying molecular mechanisms that govern pant autophagy.

### Nt acetylation and autophagy

Nt acetylation occurs on the first amino acid residues of most eukaryotic proteins and is carried out by N-terminal acetyltransferases (NATs) (Linster and Wirtz 2018; Deng and Marmorstein 2021; Shen et al. 2021b). Unlike lysine acetylation, the cellular functions and mechanisms of Nt acetylation in autophagy regulation remain elusive. Recent research has identified yeast Nat3 as an essential NAT involved in autophagy. Nat3-mediated Nt acetylation of the actin cytoskeleton constituent Act1 and the dynamin-like GTPase Vps1 (vacuolar protein sorting 1) facilitates the trafficking of Atg9 vesicles and autophagosome-vacuole fusion, respectively (Shen et al. 2021b). These findings underscore the critical roles of Nt acetylation in both upstream and downstream steps of autophagy. Notably, restoring Nt-acetylation of Act1 and Vps1 did not restore autophagy in Nat3-deficient cells (Shen et al. 2021b), suggesting that other autophagic components may also be substrates of Nat3. Since Nt-acetylation converts the charged protein N-terminus into a hydrophobic segment and has been implicated in regulating lipid-packing perturbations in membrane model systems and lipid vesicles during autophagy (Alvares et al. 2018; Shen et al. 2021b), future studies on Nt-acetylation-regulated autophagy should focus on core proteins involved in the membrane delivery and autophagosome-lysosome/vacuole fusion steps.

### Concluding remarks and future perspectives

Protein acetylation plays a pivotal role in the regulation of autophagy, a fundamental cellular process crucial for maintaining cellular homeostasis. The dynamic interplay between acetylation and deacetylation of core ATGs or autophagy-related components tightly governs various stages of the autophagic process, from initiation to completion. Additionally, acetylation influences autophagy at the transcriptional level by modifying histones and TFs. Despite accumulating evidence supporting the significance of acetylation in autophagy, the precise impact of acetylation on the activities of core ATG proteins remains incompletely understood. To address this gap, further characterization of key acetylation sites on ATG proteins, as well as the identification of relevant KATs and KDACs, is essential. These insights will enhance our understanding of the intricate molecular mechanisms through which acetylation modulates autophagy. Moreover, autophagy is subject to regulation by other PTMs, such as phosphorylation and ubiquitination (McEwan and Dikic 2011; Qi et al. 2017). Investigating the dynamic interplay between acetylation and these PTMs during autophagy regulation presents both intriguing scientific challenges and opportunities. Notably, core ATG proteins are functionally conserved across species, including plants. Recent studies indicate that acetylation also regulates plant autophagy at both transcriptional and posttranslational levels. Therefore, exploring how acetylation dynamically influences the core autophagy machinery in yeast, mammals, and plants will yield theoretical insights and novel avenues for understanding the regulatory role of acetylation in autophagy.

## Data Availability

Not applicable.

## References

[CR1] Aksnes H, Ree R, Arnesen T. Co-translational, post-translational, and non-catalytic roles of N-terminal acetyltransferases. Mol Cell. 2019;73:1097–114. 30878283 10.1016/j.molcel.2019.02.007 PMC6962057

[CR2] Alvares DS, Wilke N, Ruggiero NJ. Effect of N-terminal acetylation on lytic activity and lipid-packing perturbation induced in model membranes by a mastoparan-like peptide. Biochim Biophys Acta Biomembr. 2018;1860:737–48. 29287697 10.1016/j.bbamem.2017.12.018

[CR3] Annunziata I, van de Vlekkert D, Wolf E, Finkelstein D, Neale G, Machado E, Mosca R, Campos Y, Tillman H, Roussel MF, Andrew Weesner J, Ellen Fremuth L, Qiu X, Han M, Grosveld GC, D Azzo A. MYC competes with MiT/TFE in regulating lysosomal biogenesis and autophagy through an epigenetic rheostat. Nat Commun. 2019;10:3623. 31399583 10.1038/s41467-019-11568-0 PMC6689058

[CR4] Aroca A, Yruela I, Gotor C, Bassham DC. Persulfidation of ATG18a regulates autophagy under ER stress in Arabidopsis. Proc Natl Acad Sci U S A. 2021;118:e2023604118. 33975948 10.1073/pnas.2023604118 PMC8157962

[CR5] Bánréti A, Sass M, Graba Y. The emerging role of acetylation in the regulation of autophagy. Autophagy. 2013;9:819–29. 23466676 10.4161/auto.23908 PMC3672293

[CR6] Bao J, Zheng L, Zhang Q, Li X, Zhang X, Li Z, Bai X, Zhang Z, Huo W, Zhao X, Shang S, Wang Q, Zhang C, Ji J. Deacetylation of TFEB promotes fibrillar Aβ degradation by upregulating lysosomal biogenesis in microglia. Protein Cell. 2016;7:417–33. 27209302 10.1007/s13238-016-0269-2 PMC4887328

[CR7] Bellot G, Garcia-Medina R, Gounon P, Chiche J, Roux D, Pouysségur J, Mazure NM. Hypoxia-induced autophagy is mediated through hypoxia-inducible factor induction of BNIP3 and BNIP3L via their BH3 domains. Mol Cell Biol. 2009;29:2570–81. 19273585 10.1128/MCB.00166-09 PMC2682037

[CR8] Bertaggia E, Coletto L, Sandri M. Posttranslational modifications control FoxO3 activity during denervation. Am J Physiol Cell Physiol. 2011;302:C587–96. 22094330 10.1152/ajpcell.00142.2011

[CR9] Bhattacharjee S, Bose P, Patel K, Roy SG, Gain C, Gowda H, Robertson ES, Saha A. Transcriptional and epigenetic modulation of autophagy promotes EBV oncoprotein EBNA3C induced B-cell survival. Cell Death Dis. 2018;9:605. 29789559 10.1038/s41419-018-0668-9 PMC5964191

[CR10] Bobde RC, Kumar A, Vasudevan D. Plant-specific HDT family histone deacetylases are nucleoplasmins. Plant Cell. 2022;34:4760–77. 36069647 10.1093/plcell/koac275 PMC9709999

[CR11] Bonet-Ponce L, Saez-Atienzar S, Da Casa C, Sancho-Pelluz J, Barcia JM, Martinez-Gil N, Nava E, Jordan J, Romero FJ, Galindo MF. Rotenone induces the formation of 4-hydroxynonenal aggresomes. Role of ROS-mediated tubulin hyperacetylation and autophagic flux disruption. Mol Neurobiol. 2016;53:6194–208. 26558631 10.1007/s12035-015-9509-3

[CR12] Brown AK, Webb AE. Regulation of FOXO factors in mammalian cells. In: Ghaffari S, editor. Curr Top Dev Biol. London: Academic; 2018. p. 165–92. 10.1016/bs.ctdb.2017.10.006 PMC6383790 29433737

[CR13] Chan EYW, Kir S, Tooze SA. siRNA screening of the kinome identifies ULK1 as a multidomain modulator of autophagy. J Biol Chem. 2007;282:25464–74. 17595159 10.1074/jbc.M703663200

[CR14] Chen L, Liao B, Qi H, Xie L, Huang L, Tan W, Zhai N, Yuan L, Zhou Y, Yu L, Chen Q, Shu W, Xiao S. Autophagy contributes to regulation of the hypoxia response during submergence in *Arabidopsis thaliana*. Autophagy. 2015a;11:2233–46. 26566261 10.1080/15548627.2015.1112483 PMC4835207

[CR15] Chen L, Su Z, Huang L, Xia F, Qi H, Xie L, Xiao S, Chen Q. The AMP-activated protein kinase KIN10 is involved in the regulation of autophagy in Arabidopsi*s*. Front Plant Sci. 2017;8:1201. 28740502 10.3389/fpls.2017.01201 PMC5502289

[CR16] Chen Q, Yue F, Li W, Zou J, Xu T, Huang C, Zhang Y, Song K, Huang G, Xu G, Huang H, Li J, Liu L. Potassium bisperoxo(1,10-phenanthroline)oxovanadate (bpV(phen)) induces apoptosis and pyroptosis and disrupts the P62-HDAC6 protein interaction to suppress the acetylated microtubule-dependent degradation of autophagosomes. J Biol Chem. 2015b;290:26051–8. 26363065 10.1074/jbc.M115.653568 PMC4646258

[CR17] Chen X, Lu L, Mayer KS, Scalf M, Qian S, Lomax A, Smith LM, Zhong X. POWERDRESS interacts with HISTONE DEACETYLASE 9 to promote aging in Arabidopsis. Elife. 2016;5:e17214. 27873573 10.7554/eLife.17214 PMC5119886

[CR18] Cheng X, Ma X, Zhu Q, Song D, Ding X, Li L, Jiang X, Wang X, Tian R, Su H, Shen Z, Chen S, Liu T, Gong W, Liu W, Sun Q. Pacer is a mediator of mTORC1 and GSK3-TIP60 signaling in regulation of autophagosome maturation and lipid metabolism. Mol Cell. 2019;73:788–802. 30704899 10.1016/j.molcel.2018.12.017

[CR19] Cheng X, Sun Q. RUBCNL/Pacer and RUBCN/Rubicon in regulation of autolysosome formation and lipid metabolism. Autophagy. 2019;15:1120–1. 30894088 10.1080/15548627.2019.1596500 PMC6526810

[CR20] Cheong H, Nair U, Geng J, Klionsky DJ. The Atg1 kinase complex is involved in the regulation of protein recruitment to initiate sequestering vesicle formation for nonspecific autophagy in *Saccharomyces cerevisiae*. Mol Biol Cell. 2007;19:668–81. 18077553 10.1091/mbc.E07-08-0826 PMC2230592

[CR21] Choudhary C, Kumar C, Gnad F, Nielsen ML, Rehman M, Walther TC, Olsen JV, Mann M. Lysine acetylation targets protein complexes and co-regulates major cellular functions. Science. 2009;325:834–40. 19608861 10.1126/science.1175371

[CR22] Choudhary C, Weinert BT, Nishida Y, Verdin E, Mann M. The growing landscape of lysine acetylation links metabolism and cell signalling. Nat Rev Mol Cell Bio. 2014;15:536–50. 25053359 10.1038/nrm3841

[CR23] Deng S, Marmorstein R. Protein N-terminal acetylation: structural basis, mechanism, versatility, and regulation. Trends Biochem Sci. 2021;46:15–27. 32912665 10.1016/j.tibs.2020.08.005 PMC7749037

[CR24] Drazic A, Myklebust LM, Ree R, Arnesen T. The world of protein acetylation. Biochim Biophys Acta. 2016;1864:1372–401. 27296530 10.1016/j.bbapap.2016.06.007

[CR25] Du Y, Guo H, Guo L, Miao J, Ren H, Liu K, Ren L, He J, Wang X, Chen J, Li J, Wang Y, Wang J, Huang N. The regulatory effect of acetylation of HMGN2 and H3K27 on pyocyanin-induced autophagy in macrophages by affecting Ulk1 transcription. J Cell Mol Med. 2021;25:7524–37. 34278675 10.1111/jcmm.16788 PMC8335688

[CR26] Eisenberg T, Knauer H, Schauer A, Büttner S, Ruckenstuhl C, Carmona-Gutierrez D, Ring J, Schroeder S, Magnes C, Antonacci L, Fussi H, Deszcz L, Hartl R, Schraml E, Criollo A, Megalou E, Weiskopf D, Laun P, Heeren G, Breitenbach M, Grubeck-Loebenstein B, Herker E, Fahrenkrog B, Fröhlich K, Sinner F, Tavernarakis N, Minois N, Kroemer G, Madeo F. Induction of autophagy by spermidine promotes longevity. Nat Cell Biol. 2009;11:1305–14. 19801973 10.1038/ncb1975

[CR27] Elander PH, Holla S, Sabljic I, Gutierrez-Beltran E, Willems P, Bozhkov PV, Minina EA. Interactome of Arabidopsis ATG5 suggests functions beyond autophagy. Int J Mol Sci. 2023;24:12300. 37569688 10.3390/ijms241512300 PMC10418956

[CR28] Esteves AR, Palma AM, Gomes R, Santos D, Silva DF, Cardoso SM. Acetylation as a major determinant to microtubule-dependent autophagy: Relevance to Alzheimer’s and Parkinson disease pathology. Biochim Biophys Acta Mol Basis Dis. 2019;1865:2008–23. 30572013 10.1016/j.bbadis.2018.11.014

[CR29] Fahmy AM, Labonté P. The autophagy elongation complex (ATG5-12/16L1) positively regulates HCV replication and is required for wild-type membranous web formation. Sci Rep. 2017;7:40351. 28067309 10.1038/srep40351 PMC5220323

[CR30] Fujita N, Itoh T, Omori H, Fukuda M, Noda T, Yoshimori T. The Atg16L complex specifies the site of LC3 lipidation for membrane biogenesis in autophagy. Mol Biol Cell. 2008;19:2092–100. 18321988 10.1091/mbc.E07-12-1257 PMC2366860

[CR31] Füllgrabe J, Hajji N, Joseph B. Cracking the death code: apoptosis-related histone modifications. Cell Death Differ. 2010;17:1238–43. 20467440 10.1038/cdd.2010.58

[CR32] Füllgrabe J, Klionsky DJ, Joseph B. The return of the nucleus: transcriptional and epigenetic control of autophagy. Nat Rev Mol Cell Bio. 2014;15:65–74. 24326622 10.1038/nrm3716

[CR33] Füllgrabe J, Lynch-Day MA, Heldring N, Li W, Struijk RB, Ma Q, Hermanson O, Rosenfeld MG, Klionsky DJ, Joseph B. The histone H4 lysine 16 acetyltransferase hMOF regulates the outcome of autophagy. Nature. 2013;500:468–71. 23863932 10.1038/nature12313 PMC4006103

[CR34] Funderburk SF, Wang QJ, Yue Z. The Beclin 1–VPS34 complex–at the crossroads of autophagy and beyond. Trends Cell Biol. 2010;20:355–62. 20356743 10.1016/j.tcb.2010.03.002 PMC3781210

[CR35] Geeraert C, Ratier A, Pfisterer SG, Perdiz D, Cantaloube I, Rouault A, Pattingre S, Proikas-Cezanne T, Codogno P, Poüs C. Starvation-induced hyperacetylation of tubulin is required for the stimulation of autophagy by nutrient deprivation. J Biol Chem. 2010;285:24184–94. 20484055 10.1074/jbc.M109.091553 PMC2911293

[CR36] Grunstein M. Histone acetylation in chromatin structure and transcription. Nature. 1997;389:349–52. 9311776 10.1038/38664

[CR37] Han LL, Jia L, Wu F, Huang C. Sirtuin6 (SIRT6) Promotes the EMT of hepatocellular carcinoma by stimulating autophagic degradation of e-cadherin. Mol Cancer Res. 2019;17:2267–80. 31551254 10.1158/1541-7786.MCR-19-0321

[CR38] Hariharan N, Maejima Y, Nakae J, Paik J, DePinho RA, Sadoshima J. Deacetylation of FoxO by Sirt1 plays an essential role in mediating starvation-induced autophagy in cardiac myocytes. Circ Res. 2010;107:1470–82. 20947830 10.1161/CIRCRESAHA.110.227371 PMC3011986

[CR39] Hosokawa N, Sasaki T, Iemura S, Natsume T, Hara T, Mizushima N. Atg101, a novel mammalian autophagy protein interacting with Atg13. Autophagy. 2009;5:973–9. 19597335 10.4161/auto.5.7.9296

[CR40] Huang H, Ouyang Q, Mei K, Liu T, Sun Q, Liu W, Liu R. Acetylation of SCFD1 regulates SNARE complex formation and autophagosome-lysosome fusion. Autophagy. 2023;19:189–203. 35465820 10.1080/15548627.2022.2064624 PMC9809933

[CR41] Huang L, Wen X, Jin L, Han H, Guo H. HOOKLESS1 acetylates AUTOPHAGY-RELATED PROTEIN18a to promote autophagy during nutrient starvation in Arabidopsis. Plant Cell. 2024;36:136–57. 10.1093/plcell/koad252 PMC10734606 37823521

[CR42] Huang L, Yu L, Zhang X, Fan B, Wang F, Dai Y, Qi H, Zhou Y, Xie L, Xiao S. Autophagy regulates glucose-mediated root meristem activity by modulating ROS production in Arabidopsis. Autophagy. 2019;15:407–22. 30208757 10.1080/15548627.2018.1520547 PMC6351127

[CR43] Huang R, Xu Y, Wan W, Shou X, Qian J, You Z, Liu B, Chang C, Zhou T, Lippincott-Schwartz J, Liu W. Deacetylation of nuclear LC3 drives autophagy initiation under starvation. Mol Cell. 2015;57:456–66. 25601754 10.1016/j.molcel.2014.12.013

[CR44] Hubbert C, Guardiola A, Shao R, Kawaguchi Y, Ito A, Nixon A, Yoshida M, Wang X, Yao T. HDAC6 is a microtubule-associated deacetylase. Nature. 2002;417:455–8. 12024216 10.1038/417455a

[CR45] Itakura E, Kishi-Itakura C, Mizushima N. The hairpin-type tail-anchored SNARE syntaxin 17 targets to autophagosomes for fusion with endosomes/lysosomes. Cell. 2012;151:1256–69. 23217709 10.1016/j.cell.2012.11.001

[CR46] Iwata A, Riley BE, Johnston JA, Kopito RR. HDAC6 and microtubules are required for autophagic degradation of aggregated huntingtin. J Biol Chem. 2005;280:40282–92. 16192271 10.1074/jbc.M508786200

[CR47] Jeon M, Park J, Yang E, Baek HJ, Kim H. Regulation of autophagy by protein methylation and acetylation in cancer. J Cell Physiol. 2022;237:13–28. 34237149 10.1002/jcp.30502

[CR48] Jiang P, Nishimura T, Sakamaki Y, Itakura E, Hatta T, Natsume T, Mizushima N. The HOPS complex mediates autophagosome–lysosome fusion through interaction with syntaxin 17. Mol Biol Cell. 2014;25:1327–37. 24554770 10.1091/mbc.E13-08-0447 PMC3982997

[CR49] Jiang X, Huang Y, Liang X, Jiang F, He Y, Li T, Xu G, Zhao H, Yang W, Jiang G, Su Z, Jiang L, Liu L. Metastatic prostate cancer-associated P62 inhibits autophagy flux and promotes epithelial to mesenchymal transition by sustaining the level of HDAC6. Prostate. 2018;78:426–34. 29383752 10.1002/pros.23487 PMC5897115

[CR50] Jung CH, Jun CB, Ro S, Kim Y, Otto NM, Cao J, Kundu M, Kim D. ULK-Atg13-FIP200 complexes mediate mTOR signaling to the autophagy machinery. Mol Biol Cell. 2009;20:1992–2003. 19225151 10.1091/mbc.E08-12-1249 PMC2663920

[CR51] Kim J, Kundu M, Viollet B, Guan K. AMPK and mTOR regulate autophagy through direct phosphorylation of Ulk1. Nat Cell Biol. 2011;13:132–41. 21258367 10.1038/ncb2152 PMC3987946

[CR52] Köchl R, Hu XW, Chan EYW, Tooze SA. Microtubules facilitate autophagosome formation and fusion of autophagosomes with endosomes. Traffic. 2006;7:129–45. 16420522 10.1111/j.1600-0854.2005.00368.x

[CR53] Kotani T, Kirisako H, Koizumi M, Ohsumi Y, Nakatogawa H. The Atg2-Atg18 complex tethers pre-autophagosomal membranes to the endoplasmic reticulum for autophagosome formation. Proc Natl Acad Sci U S A. 2018;115:10363–8. 30254161 10.1073/pnas.1806727115 PMC6187169

[CR54] Kume S, Uzu T, Horiike K, Chin-Kanasaki M, Isshiki K, Araki S, Sugimoto T, Haneda M, Kashiwagi A, Koya D. Calorie restriction enhances cell adaptation to hypoxia through Sirt1-dependent mitochondrial autophagy in mouse aged kidney. J Clin Invest. 2010;120:1043–55. 20335657 10.1172/JCI41376 PMC2846062

[CR55] Lee IH, Cao L, Mostoslavsky R, Lombard DB, Liu J, Bruns NE, Tsokos M, Alt FW, Finkel T. A role for the NAD-dependent deacetylase Sirt1 in the regulation of autophagy. Proc Natl Acad Sci U S A. 2008;105:3374–9. 18296641 10.1073/pnas.0712145105 PMC2265142

[CR56] Lee IH, Finkel T. Regulation of autophagy by the p300 acetyltransferase. J Biol Chem. 2009;284:6322–8. 19124466 10.1074/jbc.M807135200 PMC5405322

[CR57] Lee IH, Kawai Y, Fergusson MM, Rovira II, Bishop AJR, Motoyama N, Cao L, Finkel T. Atg7 modulates p53 activity to regulate cell cycle and survival during metabolic stress. Science. 2012;336:225–8. 22499945 10.1126/science.1218395 PMC4721513

[CR58] Lee J, Koga H, Kawaguchi Y, Tang W, Wong E, Gao Y, Pandey UB, Kaushik S, Tresse E, Lu J, Taylor JP, Cuervo AM, Yao T. HDAC6 controls autophagosome maturation essential for ubiquitin-selective quality-control autophagy. EMBO J. 2010;29:969–80. 20075865 10.1038/emboj.2009.405 PMC2837169

[CR59] Li F, Chung T, Vierstra RD. AUTOPHAGY-RELATED11 plays a critical role in general autophagy- and senescence-induced mitophagy in Arabidopsis. Plant Cell. 2014;26:788–807. 24563201 10.1105/tpc.113.120014 PMC3967041

[CR60] Li F, Vierstra RD. Autophagy: a multifaceted intracellular system for bulk and selective recycling. Trends Plant Sci. 2012;17:526–37. 22694835 10.1016/j.tplants.2012.05.006

[CR61] Li X, Wang Y, Xiong Y, Wu J, Ding H, Chen X, Lan L, Zhang H. Galangin induces autophagy via deacetylation of LC3 by SIRT1 in HepG2 cells. Sci Rep. 2016;6:30496. 27460655 10.1038/srep30496 PMC4962058

[CR62] Li X, Yu W, Qian X, Xia Y, Zheng Y, Lee J, Li W, Lyu J, Rao G, Zhang X, Qian C, Rozen SG, Jiang T, Lu Z. Nucleus-translocated ACSS2 promotes gene transcription for lysosomal biogenesis and autophagy. Mol Cell. 2017;66:684–97. 28552616 10.1016/j.molcel.2017.04.026 PMC5521213

[CR63] Li Y, Xu X, Qi G, Cui D, Huang C, Sui X, Li G, Fan Q. Mechanisms of autophagy function and regulation in plant growth, development, and response to abiotic stress. Crop J. 2023;11:1611–25.

[CR64] Li Z, Liu X, Ma J, Zhang T, Gao X, Liu L. hnRNPK modulates selective quality-control autophagy by downregulating the expression of HDAC6 in 293 cells. Int J Oncol. 2018;53:2200–12. 30106132 10.3892/ijo.2018.4517

[CR65] Liang J, Shao SH, Xu Z, Hennessy B, Ding Z, Larrea M, Kondo S, Dumont DJ, Gutterman JU, Walker CL. The energy sensing LKB1–AMPK pathway regulates p27kip1 phosphorylation mediating the decision to enter autophagy or apoptosis. Nat Cell Biol. 2007;9:218–24. 17237771 10.1038/ncb1537

[CR66] Liang T, Qi C, Lai Y, Xie J, Wang H, Zhang L, Lin T, Jv M, Li J, Wang Y, Zhang Y, Chen Z, Qiu X, Li R, Li Z, Ye Z, Liu S, Liang X, Shi W, Wang W. HDAC6-mediated alpha-tubulin deacetylation suppresses autophagy and enhances motility of podocytes in diabetic nephropathy. J Cell Mol Med. 2020;24:11558–72. 32885602 10.1111/jcmm.15772 PMC7576268

[CR67] Lin S, Li TY, Liu Q, Zhang C, Li X, Chen Y, Zhang S, Lian G, Liu Q, Ruan K, Wang Z, Zhang C, Chien K, Wu J, Li Q, Han J, Lin S. GSK3-TIP60-ULK1 signaling pathway links growth factor deprivation to autophagy. Science. 2012;336:477–81. 22539723 10.1126/science.1217032

[CR68] Linster E, Wirtz M. N-terminal acetylation: an essential protein modification emerges as an important regulator of stress responses. J Exp Bot. 2018;69:4555–68. 29945174 10.1093/jxb/ery241

[CR69] Liu J, Kang Y, Yin S, Chen A, Wu J, Liang H, Shao L. Key role of microtubule and its acetylation in a zinc oxide nanoparticle–mediated lysosome–autophagy system. Small. 2019;15:1901073. 10.1002/smll.201901073 31062916

[CR70] Liu X, Klionsky DJ. TP53INP2/DOR protein chaperones deacetylated nuclear LC3 to the cytoplasm to promote macroautophagy. Autophagy. 2015;11:1441–2. 26213321 10.1080/15548627.2015.1074373 PMC4590662

[CR71] Liu Y, Burgos JS, Yan D, Srivastava R, Bassham H. Degradation of the endoplasmic reticulum by autophagy during ER stress in plants. Plant Cell. 2012;24:4635–51. 23175745 10.1105/tpc.112.101535 PMC3531857

[CR72] Lum JJ, Bauer DE, Kong M, Harris MH, Li C, Lindsten T, Thompson CB. Growth factor regulation of autophagy and cell survival in the absence of apoptosis. Cell. 2005;120:237–48. 15680329 10.1016/j.cell.2004.11.046

[CR73] Ma X, Wang Q. Short-chain fatty aacids attenuate renal fibrosis and enhance autophagy of renal tubular cells in diabetic mice through the HDAC2/ULK1 axis. Enm. 2022;37:432–43. 35574586 10.3803/EnM.2021.1336 PMC9262686

[CR74] Mackeh R, Lorin S, Ratier A, Mejdoubi-Charef N, Baillet A, Bruneel A, Hamaï A, Codogno P, Poüs C, Perdiz D. Reactive oxygen species, AMP-activated protein kinase, and the transcription cofactor p300 regulate α-tubulin acetyltransferase-1 (αTAT-1/MEC-17)-dependent microtubule hyperacetylation during cell Stress. J Biol Chem. 2014;289:11816–28. 24619423 10.1074/jbc.M113.507400 PMC4002089

[CR75] Majora M, Sondenheimer K, Knechten M, Uthe I, Esser C, Schiavi A, Ventura N, Krutmann J. HDAC inhibition improves autophagic and lysosomal function to prevent loss of subcutaneous fat in a mouse model of Cockayne syndrome. Sci Transl Med. 2018;10:eaam7510. 30158153 10.1126/scitranslmed.aam7510

[CR76] Mammucari C, Milan G, Romanello V, Masiero E, Rudolf R, Del Piccolo P, Burden SJ, Di Lisi R, Sandri C, Zhao J, Goldberg AL, Schiaffino S, Sandri M. FoxO3 controls autophagy in skeletal muscle in vivo. Cell Metab. 2007;6:458–71. 18054315 10.1016/j.cmet.2007.11.001

[CR77] Marshall RS, Vierstra RD. Autophagy: the master of bulk and selective recycling. Annu Rev Plant Biol. 2018;69:173–208. 29539270 10.1146/annurev-arplant-042817-040606

[CR78] Maskey D, Yousefi S, Schmid I, Zlobec I, Perren A, Friis R, Simon H. ATG5 is induced by DNA-damaging agents and promotes mitotic catastrophe independent of autophagy. Nat Commun. 2013;4:2130. 23945651 10.1038/ncomms3130 PMC3753548

[CR79] Matsunaga K, Saitoh T, Tabata K, Omori H, Satoh T, Kurotori N, Maejima I, Shirahama-Noda K, Ichimura T, Isobe T, Akira S, Noda T, Yoshimori T. Two Beclin 1-binding proteins, Atg14L and Rubicon, reciprocally regulate autophagy at different stages. Nat Cell Biol. 2009;11:385–96. 19270696 10.1038/ncb1846

[CR80] Matsuzaki H, Daitoku H, Hatta M, Aoyama H, Yoshimochi K, Fukamizu A. Acetylation of Foxo1 alters its DNA-binding ability and sensitivity to phosphorylation. Proc Natl Acad Sci U S A. 2005;102:11278–83. 16076959 10.1073/pnas.0502738102 PMC1183558

[CR81] McEwan DG, Dikic I. The three musketeers of autophagy: phosphorylation, ubiquitylation and acetylation. Trends Cell Biol. 2011;21:195–201. 21277210 10.1016/j.tcb.2010.12.006 PMC3714536

[CR82] Mercer CA, Kaliappan A, Dennis PB. A novel, human Atg13 binding protein, Atg101, interacts with ULK1 and is essential for macroautophagy. Autophagy. 2009;5:649–62. 19287211 10.4161/auto.5.5.8249

[CR83] Mizushima N. The role of the Atg1/ULK1 complex in autophagy regulation. Curr Opin Cell Biol. 2010;22:132–9. 20056399 10.1016/j.ceb.2009.12.004

[CR84] Monastyrska I, Rieter E, Klionsky DJ, Reggiori F. Multiple roles of the cytoskeleton in autophagy. Biol Rev. 2009;84:431–48. 19659885 10.1111/j.1469-185X.2009.00082.x PMC2831541

[CR85] Nair U, Jotwani A, Geng J, Gammoh N, Richerson D, Yen WL, Griffith J, Nag S, Wang K, Moss T, Baba M, McNew JA, Jiang X, Reggiori F, Melia TJ, Klionsky DJ. SNARE proteins are required for macroautophagy. Cell. 2011;146:290–302. 21784249 10.1016/j.cell.2011.06.022 PMC3143362

[CR86] Nie T, Yang S, Ma H, Zhang L, Lu F, Tao K, Wang R, Yang R, Huang L, Mao Z, Yang Q. Regulation of ER stress-induced autophagy by GSK3β-TIP60-ULK1 pathway. Cell Death Dis. 2016;7: e2563. 28032867 10.1038/cddis.2016.423 PMC5260977

[CR87] North BJ, Marshall BL, Borra MT, Denu JM, Verdin E. The human Sir2 ortholog, SIRT2, is an NAD+-dependent tubulin deacetylase. Mol Cell. 2003;11:437–44. 12620231 10.1016/s1097-2765(03)00038-8

[CR88] Nowosad A, Creff J, Jeannot P, Culerrier R, Codogno P, Manenti S, Nguyen L, Besson A. p27 controls autophagic vesicle trafficking in glucose-deprived cells via the regulation of ATAT1-mediated microtubule acetylation. Cell Death Dis. 2021;12:481. 33986251 10.1038/s41419-021-03759-9 PMC8119952

[CR89] Ohsumi Y. Molecular dissection of autophagy: two ubiquitin-like systems. Nat Rev Mol Cell Bio. 2001;2:211–6. 11265251 10.1038/35056522

[CR90] Pang J, Xiong H, Ou Y, Yang H, Xu Y, Chen S, Lai L, Ye Y, Su Z, Lin H, Huang Q, Xu X, Zheng Y. SIRT1 protects cochlear hair cell and delays age-related hearing loss via autophagy. Neurobiol Aging. 2019;80:127–37. 31170533 10.1016/j.neurobiolaging.2019.04.003

[CR91] Pehar M, Jonas MC, Hare TM, Puglielli L. SLC33A1/AT-1 protein regulates the induction of autophagy downstream of IRE1/XBP1 pathway. J Biol Chem. 2012;287:29921–30. 22787145 10.1074/jbc.M112.363911 PMC3436137

[CR92] Phadwal K, Kurian D, Salamat MKF, MacRae VE, Diack AB, Manson JC. Spermine increases acetylation of tubulins and facilitates autophagic degradation of prion aggregates. Sci Rep. 2018;8:10004. 29968775 10.1038/s41598-018-28296-y PMC6030104

[CR93] Piperno G, LeDizet M, Chang XJ. Microtubules containing acetylated alpha-tubulin in mammalian cells in culture. J Cell Biol. 1987;104:289–302. 2879846 10.1083/jcb.104.2.289 PMC2114420

[CR94] Qi H, Lei X, Wang Y, Yu S, Liu T, Zhou S, Chen J, Chen Q, Qiu R, Jiang L, Xiao S. 14-3-3 proteins contribute to autophagy by modulating SINAT-mediated degradation of ATG13. Plant Cell. 2022;34:4857–76. 36053201 10.1093/plcell/koac273 PMC9709989

[CR95] Qi H, Li J, Xia F, Chen J, Lei X, Han M, Xie L, Zhou Q, Xiao S. Arabidopsis SINAT proteins control autophagy by mediating ubiquitylation and degradation of ATG13. Plant Cell. 2020;32:263–84. 31732704 10.1105/tpc.19.00413 PMC6961628

[CR96] Qi H, Xia F, Xiao S. Autophagy in plants: physiological roles and post-translational regulation. J Integr Plant Biol. 2021;63:161–79. 32324339 10.1111/jipb.12941

[CR97] Qi H, Xia F, Xie L, Yu L, Chen Q, Zhuang X, Wang Q, Li F, Jiang L, Xie Q, Xiao S. TRAF family proteins regulate autophagy dynamics by modulating AUTOPHAGY PROTEIN6 stability in Arabidopsis. Plant Cell. 2017;29:890–911. 28351989 10.1105/tpc.17.00056 PMC5435438

[CR98] Qin L, Berk M, Chung Y, Cui D, Zhu Z, Chakraborty AA, Sharifi N. Chronic hypoxia stabilizes 3βHSD1 via autophagy suppression. Cell Rep. 2024;43:113575. 38181788 10.1016/j.celrep.2023.113575 PMC10851248

[CR99] Reggiori F, Tucker KA, Stromhaug PE, Klionsky DJ. The Atg1-Atg13 complex regulates Atg9 and Atg23 retrieval transport from the pre-autophagosomal structure. Dev Cell. 2004;6:79–90. 14723849 10.1016/s1534-5807(03)00402-7

[CR100] Salih DA, Brunet A. FoxO transcription factors in the maintenance of cellular homeostasis during aging. Curr Opin Cell Biol. 2008;20:126–36. 18394876 10.1016/j.ceb.2008.02.005 PMC2387118

[CR101] Schaaf MBE, Keulers TG, Vooijs MA, Rouschop KMA. LC3/GABARAP family proteins: autophagy-(un)related functions. FASEB J. 2016;30:3961–78. 27601442 10.1096/fj.201600698R

[CR102] Sengupta A, Molkentin JD, Yutzey KE. FoxO transcription factors promote autophagy in cardiomyocytes. J Biol Chem. 2009;284:28319–31. 19696026 10.1074/jbc.M109.024406 PMC2788882

[CR103] Seok S, Fu T, Choi S, Li Y, Zhu R, Kumar S, Sun X, Yoon G, Kang Y, Zhong W, Ma J, Kemper B, Kemper JK. Transcriptional regulation of autophagy by an FXR–CREB axis. Nature. 2014;516:108–11. 25383523 10.1038/nature13949 PMC4257899

[CR104] Settembre C, Di Malta C, Polito VA, Arencibia MG, Vetrini F, Erdin S, Erdin SU, Huynh T, Medina D, Colella P, Sardiello M, Rubinsztein DC, Ballabio A. TFEB links autophagy to lysosomal biogenesis. Science. 2011;332:1429–33. 21617040 10.1126/science.1204592 PMC3638014

[CR105] Shen Q, Shi Y, Liu J, Su H, Huang J, Zhang Y, Peng C, Zhou T, Sun Q, Wan W, Liu W. Acetylation of STX17 (syntaxin 17) controls autophagosome maturation. Autophagy. 2021a;17:1157–69. 32264736 10.1080/15548627.2020.1752471 PMC8143222

[CR106] Shen T, Jiang L, Wang X, Xu Q, Han L, Liu S, Huang T, Li H, Dai L, Li H, Lu K. Function and molecular mechanism of N-terminal acetylation in autophagy. Cell Rep. 2021b;37:109937. 34788606 10.1016/j.celrep.2021.109937

[CR107] Shu F, Xiao H, Li Q, Ren X, Liu Z, Hu B, Wang H, Wang H, Jiang G. Epigenetic and post-translational modifications in autophagy: biological functions and therapeutic targets. Signal Transduct Tar. 2023;8:32. 10.1038/s41392-022-01300-8 PMC9842768 36646695

[CR108] Son SM, Park SJ, Fernandez-Estevez M, Rubinsztein DC. Autophagy regulation by acetylation-implications for neurodegenerative diseases. Exp Mol Med. 2021;53:30–41. 33483607 10.1038/s12276-021-00556-4 PMC8080689

[CR109] Song T, Su H, Yin W, Wang L, Huang R. Acetylation modulates LC3 stability and cargo recognition. Febs Lett. 2019;593:414–22. 30633346 10.1002/1873-3468.13327

[CR110] Su H, Yang F, Wang Q, Shen Q, Huang J, Peng C, Zhang Y, Wan W, Wong CCL, Sun Q, Wang F, Zhou T, Liu W. VPS34 acetylation controls its lipid kinase activity and the initiation of canonical and non-canonical autophagy. Mol Cell. 2017;67:907–21. 28844862 10.1016/j.molcel.2017.07.024

[CR111] Sun J, Tai S, Tang L, Yang H, Chen M, Xiao Y, Li X, Zhu Z, Zhou S. Acetylation modification during autophagy and vascular aging. Front Physiol. 2021;12:598267. 33828486 10.3389/fphys.2021.598267 PMC8019697

[CR112] Sun L, Xiong H, Chen L, Dai X, Yan X, Wu Y, Yang M, Shan M, Li T, Yao J, Jiang W, He H, He F, Lian J. Deacetylation of ATG4B promotes autophagy initiation under starvation. Sci Adv. 2022;8:eabo412. 10.1126/sciadv.abo0412 PMC9348796 35921421

[CR113] Sun T, Li X, Zhang P, Chen W, Zhang H, Li D, Deng R, Qian X, Jiao L, Ji J, Li Y, Wu R, Yu Y, Feng G, Zhu X. Acetylation of Beclin 1 inhibits autophagosome maturation and promotes tumour growth. Nat Commun. 2015;6:7215. 26008601 10.1038/ncomms8215 PMC4455096

[CR114] Sun T, Jiao L, Wang Y, Yu Y, Ming L. SIRT1 induces epithelial-mesenchymal transition by promoting autophagic degradation of E-cadherin in melanoma cells. Cell Death Dis. 2018;9:136. 29374154 10.1038/s41419-017-0167-4 PMC5833732

[CR115] Suttangkakul A, Li F, Chung T, Vierstra RD. The ATG1/ATG13 protein kinase complex is both a regulator and a target of autophagic recycling in Arabidopsis. Plant Cell. 2011;23:3761–79. 21984698 10.1105/tpc.111.090993 PMC3229148

[CR116] Wang R, Tan J, Chen T, Han H, Tian R, Tan Y, Wu Y, Cui J, Chen F, Li J, Lv L, Guan X, Shang S, Lu J, Zhang Z. ATP13A2 facilitates HDAC6 recruitment to lysosome to promote autophagosome-lysosome fusion. J Cell Biol. 2019;218:267–84. 30538141 10.1083/jcb.201804165 PMC6314552

[CR117] Wang W, Wang Q, Wan D, Sun Y, Wang L, Chen H, Liu C, Petersen RB, Li J, Xue W, Zheng L, Huang K. Histone HIST1H1C/H1.2 regulates autophagy in the development of diabetic retinopathy. Autophagy. 2017;13:941–54. 28409999 10.1080/15548627.2017.1293768 PMC5446066

[CR118] Wang Y, Huang Y, Liu J, Zhang J, Xu M, You Z, Peng C, Gong Z, Liu W. Acetyltransferase GCN5 regulates autophagy and lysosome biogenesis by targeting TFEB. Embo Rep. 2020;21:e48335. 31750630 10.15252/embr.201948335 PMC6945067

[CR119] Wang Z, Miao G, Xue X, Guo X, Yuan C, Wang Z, Zhang G, Chen Y, Feng D, Hu J, Zhang H. The Vici syndrome protein EPG5 is a Rab7 effector that determines the fusion specificity of autophagosomes with late endosomes/lysosomes. Mol Cell. 2016;63:781–95. 27588602 10.1016/j.molcel.2016.08.021

[CR120] Weinert BT, Schölz C, Wagner SA, Iesmantavicius V, Su D, Daniel JA, Choudhary C. Lysine succinylation is a frequently occurring modification in prokaryotes and eukaryotes and extensively overlaps with acetylation. Cell Rep. 2013;4:842–51. 23954790 10.1016/j.celrep.2013.07.024

[CR121] Wen X, Klionsky DJ. An overview of macroautophagy in yeast. J Mol Biol. 2016;428:1681–99. 26908221 10.1016/j.jmb.2016.02.021 PMC4846508

[CR122] Xia L, Kong X, Song H, Han Q, Zhang S. Advances in proteome-wide analysis of plant lysine acetylation. Plant Commun. 2022;3:100266. 35059632 10.1016/j.xplc.2021.100266 PMC8760137

[CR123] Xie R, Nguyen S, McKeehan WL, Liu L. Acetylated microtubules are required for fusion of autophagosomes with lysosomes. BMC Cell Biol. 2010;11:89. 21092184 10.1186/1471-2121-11-89 PMC2995476

[CR124] Xie Y, Kang R, Sun X, Zhong M, Huang J, Klionsky DJ, Tang D. Posttranslational modification of autophagy-related proteins in macroautophagy. Autophagy. 2015;11:28–45. 25484070 10.4161/15548627.2014.984267 PMC4502723

[CR125] Xiong Y, Contento AL, Nguyen PQ, Bassham DC. Degradation of oxidized proteins by autophagy during oxidative stress in Arabidopsis. Plant Physiol. 2007;143:291–9. 17098847 10.1104/pp.106.092106 PMC1761971

[CR126] Xu Y, Wan W. Acetylation in the regulation of autophagy. Autophagy. 2023;19:379–87. 35435793 10.1080/15548627.2022.2062112 PMC9851266

[CR127] Yang C, Luo M, Zhuang X, Li F, Gao C. Transcriptional and epigenetic regulation of autophagy in plants. Trends Genet. 2020a;36:676–88. 32674948 10.1016/j.tig.2020.06.013

[CR128] Yang C, Shen W, Yang L, Sun Y, Li X, Lai M, Wei J, Wang C, Xu Y, Li F, Liang S, Yang C, Zhong S, Luo M, Gao C. HY5-HDA9 module transcriptionally regulates plant autophagy in response to light-to-dark conversion and nitrogen starvation. Mol Plant. 2020b;13:515–31. 32087368 10.1016/j.molp.2020.02.011

[CR129] Yang D, Xiao C, Long F, Su Z, Jia W, Qin M, Huang M, Wu W, Suguro R, Liu X, Zhu Y. HDAC4 regulates vascular inflammation via activation of autophagy. Cardiovasc Res. 2018;114:1016–28. 29529137 10.1093/cvr/cvy051

[CR130] Yang L, Zhao L, Cui L, Huang Y, Ye J, Zhang Q, Jiang X, Zhang D, Huang Y. Decreased alpha-tubulin acetylation induced by an acidic environment impairs autophagosome formation and leads to rat cardiomyocyte injury. J Mol Cell Cardiol. 2019;127:143–53. 30582931 10.1016/j.yjmcc.2018.12.011

[CR131] Yang X, Seto E. The Rpd3/Hda1 family of lysine deacetylases: from bacteria and yeast to mice and men. Nat Rev Mol Cell Bio. 2008;9:206–18. 18292778 10.1038/nrm2346 PMC2667380

[CR132] Yi C, Yu L. How does acetylation regulate autophagy? Autophagy. 2012;8:1529–30. 22732483 10.4161/auto.21156

[CR133] Yi C, Ma M, Ran L, Zheng J, Tong J, Zhu J, Ma C, Sun Y, Zhang S, Feng W, Zhu L, Le Y, Gong X, Yan X, Hong B, Jiang F, Xie Z, Miao D, Deng H, Yu L. Function and molecular mechanism of acetylation in autophagy regulation. Science. 2012;336:474–7. 22539722 10.1126/science.1216990

[CR134] Young ARJ, Chan EYW, Hu XW, Köchl R, Crawshaw SG, High S, Hailey DW, Lippincott-Schwartz J, Tooze SA. Starvation and ULK1-dependent cycling of mammalian Atg9 between the TGN and endosomes. J Cell Sci. 2006;119:3888–900. 16940348 10.1242/jcs.03172

[CR135] Yuan H, Rossetto D, Mellert H, Dang W, Srinivasan M, Johnson J, Hodawadekar S, Ding EC, Speicher K, Abshiru N, Perry R, Wu J, Yang C, Zheng YG, Speicher DW, Thibault P, Verreault A, Johnson FB, Berger SL, Sternglanz R, McMahon SB, Côté J, Marmorstein R. MYST protein acetyltransferase activity requires active site lysine autoacetylation. EMBO J. 2012;31:58–70. 22020126 10.1038/emboj.2011.382 PMC3252582

[CR136] Zhang B, Shao L, Wang J, Zhang Y, Guo X, Peng Y, Cao Y, Lai Z. Phosphorylation of ATG18a by BAK1 suppresses autophagy and attenuates plant resistance against necrotrophic pathogens. Autophagy. 2021;17:2093–110. 32804012 10.1080/15548627.2020.1810426 PMC8496540

[CR137] Zhang J, Wang J, Zhou Z, Park J, Wang L, Wu S, Sun X, Lu L, Wang T, Lin Q, Sze SK, Huang D, Shen H. Importance of TFEB acetylation in control of its transcriptional activity and lysosomal function in response to histone deacetylase inhibitors. Autophagy. 2018;14:1043–59. 30059277 10.1080/15548627.2018.1447290 PMC6103407

[CR138] Zhang X, Cheng X, Yu L, Yang J, Calvo R, Patnaik S, Hu X, Gao Q, Yang M, Lawas M. MCOLN1 is a ROS sensor in lysosomes that regulates autophagy. Nat Commun. 2016;7:12109. 27357649 10.1038/ncomms12109 PMC4931332

[CR139] Zhao Y, Yang J, Liao W, Liu X, Zhang H, Wang S, Wang D, Feng J, Yu L, Zhu WG. Cytosolic FoxO1 is essential for the induction of autophagy and tumour suppressor activity. Nat Cell Biol. 2010;12:665–75. 20543840 10.1038/ncb2069

[CR140] Zheng Z, Zhou Y, Ye L, Lu Q, Zhang K, Zhang J, Xie L, Wu Y, Xu K, Zhang H, Xiao J. Histone deacetylase 6 inhibition restores autophagic flux to promote functional recovery after spinal cord injury. Exp Neurol. 2020;324:113138. 31794745 10.1016/j.expneurol.2019.113138

[CR141] Zhong Y, Wang QJ, Li X, Yan Y, Backer JM, Chait BT, Heintz N, Yue Z. Distinct regulation of autophagic activity by Atg14L and Rubicon associated with Beclin 1–phosphatidylinositol-3-kinase complex. Nat Cell Biol. 2009;11:468–76. 19270693 10.1038/ncb1854 PMC2664389

[CR142] Zhuang X, Chung KP, Cui Y, Lin W, Gao C, Kang B, Jiang L. ATG9 regulates autophagosome progression from the endoplasmic reticulum in Arabidopsis. Proc Natl Acad Sci U S A. 2017;114:E426. 28053229 10.1073/pnas.1616299114 PMC5255614

